# Involvement of the Inconstant Bursa of the Fifth Metatarsophalangeal Joint in Psoriatic Arthritis: A Clinical and Ultrasonographic Study

**DOI:** 10.1155/2014/174841

**Published:** 2014-07-01

**Authors:** Giovanni Ciancio, Stefania Volpinari, Maria Fotinidi, Federica Furini, Ilaria Farina, Alessandra Bortoluzzi, Manuela Ferracin, Francesca Bandinelli, Carlo Orzincolo, Francesco Trotta, Marcello Govoni

**Affiliations:** ^1^Rheumatology Unit, Department of Medical Sciences, University of Ferrara and S. Anna University Hospital, Via Aldo Moro 8, 44124 Cona, Ferrara, Italy; ^2^Department of Morphology, Surgery and Experimental Medicine, University of Ferrara, Via Fossato di Mortara 64/b, 44121 Ferrara, Italy; ^3^Department of Internal Medicine, Rheumatology Division, University of Florence, Viale Pieraccini 18, 50139 Florence, Italy; ^4^Department of Radiology, AUSL of Ravenna, Viale Stradone 9, 48018 Ravenna, Italy

## Abstract

*Objective*. To evaluate the involvement of the bursa located next to the head of the 5th metatarsal bone in patients with psoriatic arthritis (PsA) in comparison with the other seronegative spondyloarthritis (SpA). *Methods*. All patients with PsA seen during a period of 24 months were enrolled. The control group included healthy subjects and patients with the other SpA. All subjects underwent clinical and ultrasound (US) examination of the lateral surface of the 5th metatarsal. *Results*. 150 PsA patients (88 M; 62 F), 172 SpA (107 M; 65 F), and 95 healthy controls (58 M; 37 F) were evaluated. Based on clinical and US evaluation, bursitis was diagnosed in 17/150 (11.3%) PsA patients but in none of the SpA (*P* < 0.0001) and healthy (*P* = 0.0002) controls. In detecting bursitis, US was more sensitive than clinical examination, although the difference did not reach statistical significance (*P* = 0.09). *Conclusion*. The bursa of the 5th metatarsophalangeal joint appears to be involved in PsA more frequently than by chance. If confirmed by other studies, this finding could be considered as a distinctive clinical sign of PsA, useful for differential diagnosis with the other SpA. In asymptomatic patients, US proved to be more sensitive in the detection of bursitis.

## 1. Introduction

Psoriatic arthritis (PsA) is a chronic inflammatory condition belonging to the spondyloarthritis (SpA) group. The clinical picture is very heterogenous, with a possible combination of axial manifestations, peripheral arthritis, enthesitis, dactylitis, and other musculoskeletal complaints, including bursitis [1]. The inflammatory involvement of the inconstant bursa located between the insertion of the tendon of abductor digiti minimi and the head of the 5th metatarsal bone was first described in a PsA patient in 2001 [2]. Thereafter, to the best of our knowledge, no other cases have been reported in the literature. In this study, a cohort patient has been examined in order to evaluate the prevalence of this peculiar bursitis in PsA and possibly its specificity in comparison with patients suffering from the other SpA.

## 2. Materials and Methods

All consecutive PsA patients (*de novo* cases or with established disease) attending our department throughout 24 months were enrolled. All patients satisfied CASPAR classification criteria [3]. Demographic characteristics are summarized in Table 1. As controls, patients with other SpA and a group of healthy subjects were recruited. SpA patients were diagnosed in accordance with the current criteria, that is, the modified New York criteria for ankylosing spondylitis (AS) [4], the Berlin criteria for reactive arthritis (ReA) [5], and the European Spondyloarthropathy Study Group for undifferentiated SpA (uSpA) [6]. Patients with inflammatory bowel disease associated with SpA (IBD-SpA) had to have a biopsy confirming the diagnosis of Crohn's disease or ulcerative colitis.

The coexistence of other inflammatory rheumatic conditions different from PsA and SpA, including gout or chondrocalcinosis, had to be preliminary excluded in all examined patients.

Clinical and ultrasonographic (US) evaluation of the lateral surface of the head of the 5th metatarsal bone were performed in all PsA patients and controls focusing on the detection of inconstant bursitis on this site.

Disease activity was assessed in all SpA and PsA patients in accordance with the currently available indices, that is, ASDAS (Ankylosing Spondylitis Disease Activity Score) for AS and uSpA [7, 8]; DAREA (Disease Activity Index for Reactive Arthritis) for ReA [9]; DAPSA (Disease Activity Index for Psoriatic Arthritis) and ASDAS for PsA [10, 11]. The last two scores (DAPSA and ASDAS) were also used for the assessment of IBD-SpA disease activity.

Written informed consent was obtained from all the participants and the local ethics committee approval was granted. Bursitis was clinically diagnosed if a painful soft tissue swelling, deforming the lateral surface of the head of the 5th metatarsal bone, was present.

Clinical evaluation was performed separately by two blinded rheumatologists (SV and MF) who soon after the examination compared their results. If their clinical judgment for the presence or absence of bursitis was different, they carried out together a further examination to achieve consensus. Clinical interobserver agreement was estimated based on their individual physical evaluations.

US examination of the lateral surface of the fifth metatarsal was performed by a rheumatologist experienced in musculoskeletal sonography (G.C.) who was unaware of the subjects clinical data. A Logiq5 machine (General Electric Medical Systems, Korea) equipped with a multifrequency linear array transducer, 7–12 MHz, was used. Each subject was asked to assume the supine position with the knee flexed at 45° and the foot resting on the couch. Both longitudinal and transverse scans, respectively, parallel and perpendicular to the axis of the fifth metatarsal, were performed. Bursitis was defined with a cutoff 2 mm of thickness of the bursa according to Balint et al. [12] and had to be appreciable in both scans. Vascularization of the bursa was evaluated with Power Doppler US (PDUS), standardized with pulse repetition frequency of 750 Hz and a gain of 53 dB, and scored either with a binary item (negative if absent and positive if any signal) [13] or with a semiquantitative scale from 0 to 3 (0 = absence of flow; 1 = mild, single vessel signal; 2 = moderate, confluent vessels; 3 = marked, vessel signals in over half the area of the bursa) [14]. In all subjects with US evidence of bursitis, sonographic assessment was also extended to the 5th metatarsophalangeal (MTP) joint, examined longitudinally from the dorsal view, in order to evaluate the simultaneous presence of joint inflammatory alterations (i.e., effusion, synovitis, joint erosions, and altered synovial vascularization) [14, 15].

US inter- and intraobserver agreement (presence/absence of bursitis) was estimated by recording in a digital archiving computer system the images of all patients with evidence of bursitis and the pictures of 50 other subjects randomly chosen among PsA patients and controls. The identity of PsA patients was mixed with controls. All saved images were read two months after the initial scanning by the same rheumatologist who performed US examination (GC), blind to previous results, and by a radiologist (CO) expert in musculoskeletal US.

### 2.1. Statistical Analysis

Differences between groups were examined using two-sided Fisher's exact test and relative risk calculation from GraphPad software. A *P* value <0.05 was considered statistically significant. Interobserver agreement between the clinical investigators and the inter- and intraobserver agreement for US examination were calculated using an unweighted *k* test. A *k* value <0.40 was considered low; 0.41–0.60 moderate; 0.61–0.80 good; and 0.81–1 excellent.

## 3. Results

One hundred fifty patients with PsA (88M, 62 F; mean age: 45.6 ± 14.1 yrs), 172 patients with SpA (107 M, 65 F; mean age: 50.3 ± 14.8 yrs), and 95 healthy controls (58 M; 37 F; mean age: 45.8 ± 12.6 yrs) were evaluated in the study period. The other SpA group included 47 AS, 20 IBD-SpA, 92 uSpA, and 13 ReA.

Overall, an active disease was found in 63/150 (42%) PsA and 67/172 (38.9%) SpA (*P* = 0.57).

**Figure 1:**
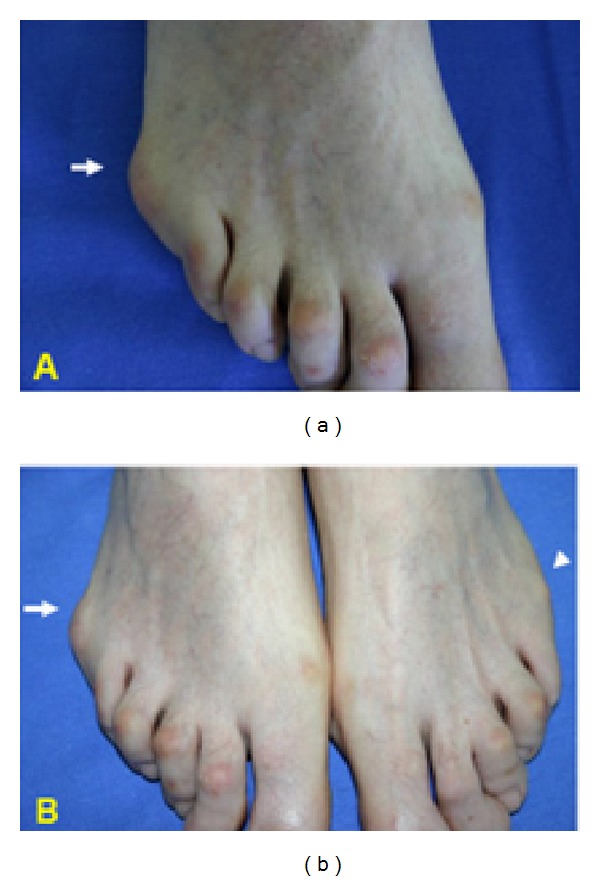
Bursitis of the 5th MTP in PsA. (a) Right foot. Marked soft tissue swelling deforming the lateral surface of the head of the 5th metatarsal bone (arrow) in a PsA patient. (b) Comparison between the right (bursitis: arrow) and the left foot (normal aspect: arrowhead) in the same patient.

**Figure 2:**
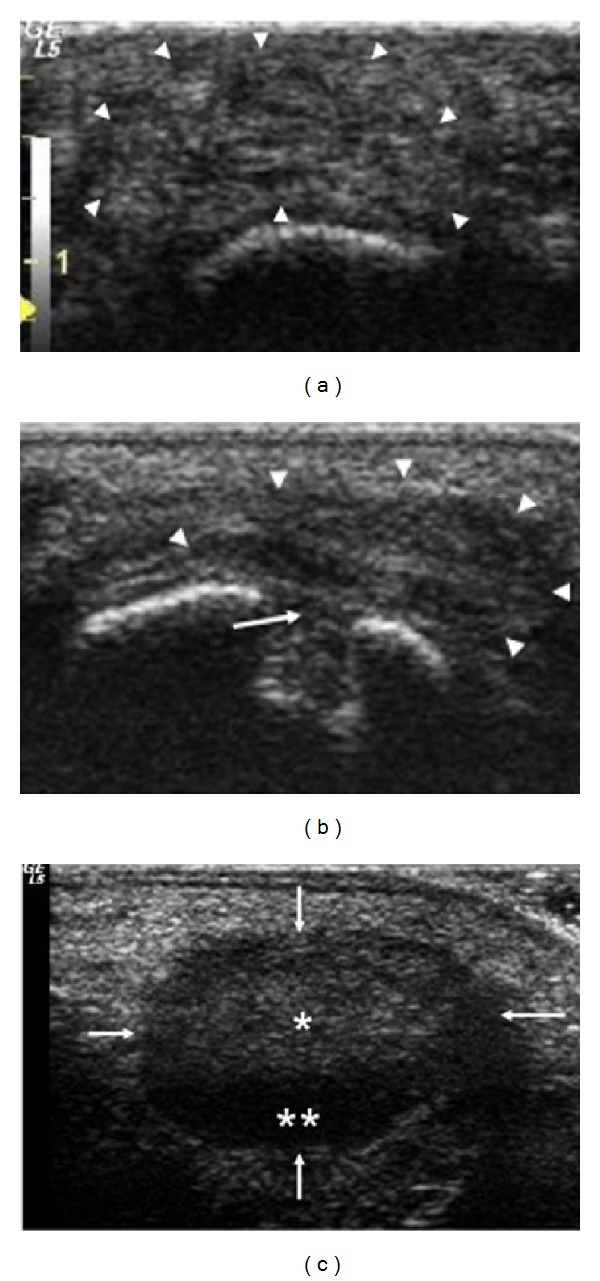
US of the lateral surface of the 5th MTP in PsA (gray-scale). (a) Left foot: presence of bursitis (arrowheads) with inhomogeneous hyperechoic pattern. (b) Wide bone erosion (arrow) of the head of the 5th metatarsal below the bursa (arrowheads). (c) Right foot: mixed hyperechoic (single white star) and hypoechoic (double white star) pattern of bursitis (arrow).

**Figure 3:**
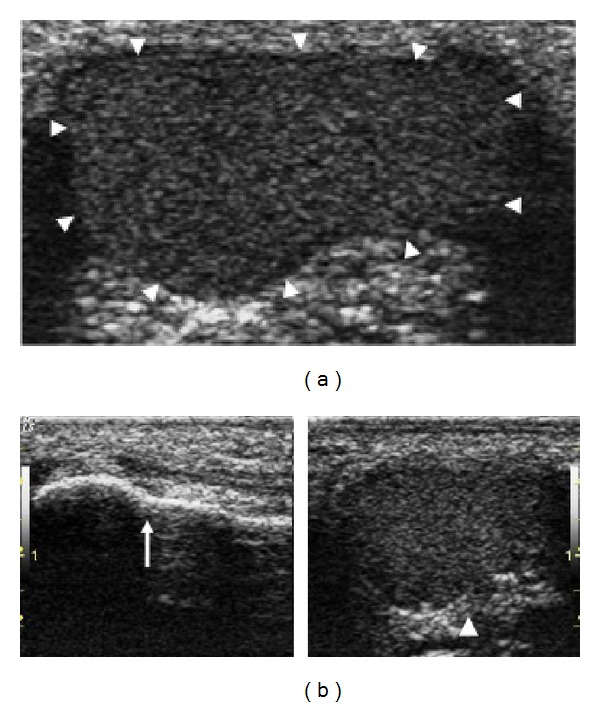
US of the lateral surface of the 5th MTP in PsA (gray-scale). (a) Left foot: evidence of wide bursitis (arrowheads) with inhomogeneous hyperechoic pattern. (b) Comparison between the right foot (arrow: normal aspect) and the left foot (arrowhead: bursitis) in the same PsA patient.

A clinical diagnosis of bursitis (Figure 1) was made in 8/150 (5.3%) PsA patients (5/8 bilaterally; 3/8 monolaterally), in none of the 95 healthy controls (*P* = 0.0245; RR: 10.8079 [0.6310 to 185.1197  95% CI]), and in none of the 172 SpA patients (*P* = 0.002; RR: 19.4768 [1.1336 to 334.6480  95% CI]). On US examination, bursitis was detected in 17/150 (11.3%) PsA patients, 8 of which corresponded to those already identified by clinical examination; in none of the healthy controls (*P* = 0.0002; RR: 22.2517 [1.3538 to 365.7418  95% CI]), and in none of the SpA group (*P* < 0.0001; RR: 40.0993 [2.4319 to 661.1959  95% CI]) (Table 2). On US examination, bursitis appeared as an irregularly oval extra-articular area of variable size adjacent to the lateral surface of the head of the 5th metatarsal bone, well distinguishable from the subcutaneous tissue. On grey-scale, three main echogenic patterns were described: (i) inhomogeneous hyperechoic pattern (9 cases); (ii) inhomogeneous hypoechoic pattern (5 cases); and (iii) mixed hyperechoic/hypoechoic pattern (3 cases) (Figures 2 and 3). PD signal inside the bursa was found in 7 patients (grade 1 in 4 cases; grade 2 in 3 cases). The presence of bone erosion on the lateral aspect of the head of the 5th metatarsal, just below the bursa, was evidenced in 9 patients (Figure 2(b)). US signs of inflammation of the 5th MTP joint were evidenced in none of the PsA patients with bursitis. In detecting bursitis, US was more sensitive than clinical examination (11.3% versus 5.3%), although the difference did not reach statistical significance (*P* = 0.09).

No differences about clinical (disease duration, radiographic damage in the feet, and BMI) or demographic (sex, age) items were recorded between PsA patients with and without bursitis.

Clinical interobserver agreement was excellent (*k* = 0.93). There was also an excellent level of intraobserver (*k* = 0.96) and interobserver (*k* = 0.87) agreement for US evidence of bursitis.

## 4. Discussion

The involvement of synovial bursae is relatively frequent in PsA [1]. However, several bursae are often inconstant, small, and poorly documented, so their involvement cannot be easily recognized even by expert clinicians [16]. Among these, an inconstant bursa is located between the tendon of abductor digiti minimi and the head of the 5th metatarsal bone [17, 18]. Its inflammatory involvement in PsA has been reported in a single case in 2001 [2], but other cases on this subject have not been described in the literature. In our cohort, clinical inspection evidenced that the involvement of this bursa is not unusual in PsA, unlike the other SpA group and healthy controls, suggesting that this could be regarded as a putative distinctive feature of PsA. In all cases, US examination confirmed the physical examination, allowing in addition a broad analysis of the structure, size, and vascular alterations of the bursa. Moreover, US proved to be more sensitive than clinical examination in detecting subclinical involvement of this bursa in PsA patients. These data confirm once more that US and PDUS are superior to clinical evaluation in detecting subclinical osteoarticular inflammatory abnormalities including enthesitis or bursitis [12–14]. In 9/17 PsA patients with bursitis, US also highlighted the simultaneous presence of bone erosion on the lateral aspect of the head of the 5th metatarsal without signs of concomitant inflammation of the 5th MTP joint. As already described [18], this peculiar bursa is contiguous to the 5th MTP joint, which can explain the development of marginal bone erosions on the head of the 5th metatarsal as a purely extra-articular event also in the absence of concurrent joint synovitis.

In the available literature, this inconstant bursa has been described in about 24% of feet [17]; therefore, the number of PsA patients with bursitis which we have found in our series can be considered relatively high. However, it is necessary to emphasize the special attention given in detecting this uncommon bursitis in our work and above all the sensitivity of US examination, which is not always routinely used in clinical practice.

Given the importance of enthesitis in the pathogenesis of SpA and with reference to the concept of “enthesis organ” theory, a potential limitation of our study that might be considered is the lack of a simultaneous systematic US study of enthesis at the attachment of the abductor digiti minimi to the 5th metatarsal bone [19]. However, our protocol planned to focus exclusively on the presence or absence of bursitis because of its peculiarity and its inconstant presence, without investigating the insertion of the tendon. A study including also systematic US evaluation of enthesitis of the abductor digiti minimi could be a reasonable future step on this topic.

In conclusion, the inconstant bursa of the 5th MTP joint appears to be involved in PsA more frequently than by chance. Given the importance of trauma in the pathogenesis of PsA, it could be hypothesized that repeated microtrauma in this anatomical site of the foot (including the conflict with the shoe) may favor the onset of bursitis in these patients.

If confirmed by other studies on a larger number of patients, this peculiar bursitis could be considered as a distinctive sign evocative of PsA in comparison with the other SpA. Better attention to this little known bursa should be advisable especially when evaluating patients with suspected PsA in order to ensure its proper identification. US has proved to be more sensitive than clinical examination in the detection subclinical bursitis, confirming the growing role of this imaging tool in highlighting soft tissues inflammatory lesions.

## Figures and Tables

**Table 1:** Demographic characteristics of PsA patients (*n* = 150).

Age (years) (mean ± SD)	45.6 ± 14.1
Male/female	88/62
Disease duration (months) (mean ± SD)	27.5 ± 18.6

Clinical manifestations	
Peripheral arthritis (polyarthritis, oligoarthritis, and distal IF)	72%
Axial disease	25%
Enthesitis	47%
Dactylitis	38%
Cutaneous psoriasis	86%

Drug treatment	
None	12%
NSAID	20%
Low dose steroids and/or DMARDs	48%
Anti-TNF therapy	20%

NSAID: nonsteroidal anti-inflammatory drugs; DMARDs: disease-modifying antirheumatic drugs; TNF: tumor necrosis factor.

**Table 2:** Clinical and US evidence of bursitis in PsA patients, other SpA patients, and healthy controls.

	Psoriatic arthritis *n* = 150	Healthy controls *n* = 95	SpA patients *n* = 172
Clinical evidence of bursitis	8/150 (5.3%)	0/95∗ *P* = 0.0245; RR: 10.8079 [0.6310 to 185.1197 95% CI]	0/172∗ *P* = 0.002; RR: 19.4768 [1.1336 to 334.6480 95% CI]
US evidence of bursitis	17/150 (11.3%)	0/95∗ *P* = 0.0002; RR: 22.2517 [1.3538 to 365.7418 95% CI]	0/172∗ *P* < 0.0001; RR: 40.0993 [2.4319 to 661.1959 95% CI]
Clinical + US evidence of bursitis	17/150 (11.3%)	0/95∗ *P* = 0.0002; RR: 22.2517 [1.3538 to 365.7418 95% CI]	0/172∗ *P* < 0.0001; RR: 40.0993 [2.4319 to 661.1959 95% CI]

*If a group has no events (0), 0.5 has been added to all cells before calculating the relative risk to prevent division by zero.
